# The role of music therapy in palliative and end-of-life care: a narrative review of psychosocial outcomes

**DOI:** 10.3389/fpubh.2026.1884748

**Published:** 2026-06-30

**Authors:** Jared Kyle Sieusahai

**Affiliations:** School of Public Health, University of Alberta, Edmonton, AB, Canada

**Keywords:** bereavement, end-of-life care, legacy work, live music, music therapy, palliative care, psychosocial outcomes, spiritual wellbeing

## Abstract

**Background/Objectives:**

Music therapy has emerged as a valuable component of palliative and end-of-life care, addressing the physical, emotional, social, and spiritual needs of patients and their families. Despite growing clinical adoption, the mechanisms and breadth of psychosocial outcomes remain inconsistently synthesized. This narrative review aims to synthesize peer-reviewed evidence on music therapy's psychosocial outcomes in palliative settings, including mechanisms, populations, modalities, and evidence gaps.

**Methods:**

A systematic search was conducted in PubMed and the University of Alberta Library Primo discovery platform in May and June 2026, searching across PsycINFO, CINAHL, MEDLINE, Academic Search Complete, and additional databases. Primary search strings targeted music therapy in combination with palliative care, end-of-life care, and hospice settings. After screening for relevance and applying inclusion criteria, 33 peer-reviewed sources were included.

**Results:**

Evidence from randomized controlled trials, systematic reviews, and qualitative studies supports consistent improvements in pain, anxiety, mood, and subjective wellbeing. Biographical approaches involving personally meaningful songs and legacy work meaningfully enhance spiritual wellbeing and existential integration in dying patients. Music therapy benefits extend to family members and informal caregivers in the pre-bereavement phase. However, most studies are small, at high risk of bias, and heavily concentrated in Western, high-income settings. The non-Western palliative music therapy literature is critically sparse in English-language indexed databases, representing a gap in the evidence base rather than evidence of absence.

**Conclusions:**

Music therapy is a flexible, low-risk, and clinically meaningful adjunct in palliative care. Future research should prioritize large-scale, culturally sensitive trials, standardized outcome measures, and explicit investigation of music therapy across diverse religious and cultural contexts.

## 1 Introduction

Palliative care aims to improve the quality of life of patients facing life-threatening illness and their families, addressing suffering across physical, psychological, social, and spiritual dimensions (39). Within this framework, music therapy has emerged as one of the most widely used complementary interventions, offering a non-pharmacological approach to symptom management and psychosocial support at the end of life (1). Unlike curative treatments, music therapy does not seek to alter the disease trajectory; instead, it attends to the person: their emotional state, their relationships, their sense of meaning, and their dignity at a time when these dimensions of experience are most acutely felt.

The growing body of research on music therapy in palliative settings reflects increasing recognition that dying well is not only a medical event but a deeply human one. Music has a unique capacity to access emotional and biographical dimensions of experience that verbal communication often cannot reach, making it particularly well-suited to contexts where patients may have diminished physical capacity, cognitive decline, or limited energy for extended conversation (2). Across cultures and throughout human history, music has accompanied death and mourning, offering solace to the bereaved and dignity to the dying (3).

Despite promising evidence, the palliative music therapy literature faces significant methodological challenges, including small sample sizes, heterogeneous intervention designs, and a lack of standardized outcome measures (4–6). A foundational Cochrane review of music therapy for end-of-life care identified benefits for pain and anxiety but noted that most trials had high risk of bias and limited generalizability (6). Most research originates from North America, Europe, and Australia, leaving substantial gaps in understanding of music therapy's effectiveness across culturally diverse and non-Western populations (7, 8). This narrative review synthesizes the current evidence on music therapy's psychosocial outcomes in palliative and end-of-life care, with attention to mechanisms, populations, modalities, and persistent gaps.

## 2 Methods

This narrative review was conducted in accordance with standard guidance for narrative reviews (9). The review addresses the following aim: to synthesize peer-reviewed evidence on how music therapy shapes psychosocial outcomes in palliative and end-of-life care, including mechanisms, populations, modalities, family and caregiver outcomes, and evidence gaps.

### 2.1 Search strategy

A systematic literature search was conducted across two platforms in May and June 2026. First, PubMed was searched using three primary strings: “music therapy”[Title/Abstract] AND “palliative care”[Title/Abstract] (170 results); “music therapy”[Title/Abstract] AND “end-of-life”[Title/Abstract] (53 results); and “music therapy”[Title/Abstract] AND “hospice”[Title/Abstract] (70 results), yielding a combined deduplicated total of 220 results. Second, the University of Alberta Library Primo discovery platform was searched, which aggregates across PsycINFO, CINAHL, MEDLINE, Academic Search Complete, Web of Science, Scopus, and additional databases. The same three search term pairs were applied: music therapy AND palliative care (1,032 results); music therapy AND end-of-life (328 results); and music therapy AND hospice (551 results). Supplementary searches in PubMed targeted non-Western contexts, spiritual care, bereavement, and caregiver outcomes. Reference lists of all included systematic reviews and meta-analyses were hand-searched to identify additional relevant literature. Searches were conducted in English; the absence of non-Western literature identified reflects a gap in English-language indexed databases rather than an absolute absence of research in those contexts.

### 2.2 Inclusion and exclusion criteria

Studies were included if they: (1) examined music therapy as a primary intervention in palliative, hospice, or end-of-life care settings; (2) addressed psychosocial, spiritual, physical, or caregiver outcomes; (3) were published in English between 2000 and 2026; and (4) were peer-reviewed. Systematic reviews, meta-analyses, randomized controlled trials, pilot studies, qualitative studies, and mixed-methods research were all eligible. Studies were excluded if music therapy was a minor component of a broader intervention, or if they were conference abstracts or gray literature. The restriction to English-language literature is acknowledged as a limitation that may have excluded relevant non-Western research published in other languages.

### 2.3 Screening and quality assessment

Titles and abstracts of results from both search platforms were screened for relevance by the author. Full texts of relevant studies were retrieved and assessed against inclusion criteria. A total of 33 peer-reviewed sources were included. Given the narrative review format, formal risk-of-bias scoring was not applied; instead, methodological quality is assessed descriptively throughout the Results sections and summarized in Table 1, using a three-level rating: high (systematic reviews with comprehensive search strategies, Cochrane reviews, or RCTs with low risk of bias), moderate (RCTs or systematic reviews with identified limitations, mixed-methods studies with clear methodology), and low or low-moderate (pilot studies, case studies, commentaries, conceptual papers, or single-site qualitative studies). This approach is consistent with narrative review standards as described by Grant and Booth (9).

**Table 1:** Summary of included studies with quality assessment.

References	Country	Design	Sample	Population	Key findings	Quality
Gutgsell et al. (10)	USA	RCT	*n* = 200	Inpatient palliative	Single MT session significantly reduced pain vs. standard care	Moderate
Warth et al. (11)	Germany	RCT	NR	Palliative inpatients	Improved relaxation, wellbeing, fatigue; no significant pain reduction vs. verbal relaxation control	Moderate
McConnell et al. (4)	UK/Ireland	Systematic review	Multiple RCTs	End-of-life patients	MT reduced pain but no QoL improvement; all trials had high risk of bias	Moderate
McConnell and Porter (3)	UK/Ireland	Realist review	51 articles	Palliative care patients	Maps mechanisms: therapeutic relationship, individualized music, flexible methods	Moderate
McConnell et al. (5)	UK/Ireland	RCT pilot	NR	Palliative care patients	Feasibility confirmed; standardized outcome measures needed for phase III trial	Low-Moderate
Bradt and Dileo (6)	International	Cochrane review	Multiple RCTs	End-of-life patients	Benefits for pain and anxiety; all trials had significant methodological limitations	High
Peng et al. (12)	USA	Mixed methods	NR	Hospice/palliative inpatients	Live music reduced pain, anxiety, depression, nausea; increased wellbeing	Low-Moderate
Warth et al. (18)	Germany	Meta-analysis	Multiple studies	Palliative patients	Brief psychosocial interventions including MT produced small-moderate QoL improvements	Moderate
Warth et al. (16)	Germany/International	RCT protocol	NR	Palliative patients	SOL study protocol; targets spiritual wellbeing, ego-integrity, cortisol	N/A
Warth et al. (15)	Germany/International	Multicenter RCT	NR	Palliative patients and families	SOL: improved spiritual wellbeing, ego-integrity, distress vs. relaxation control	High
Koehler et al. (21)	Germany	RCT substudy	NR	Palliative patients	MT produced greater cortisol/distress reduction than mindfulness	Moderate
Schmid et al. (2)	Norway	Integrative review	Multiple studies	Patients and providers	Psychophysiological change; music links to emotional expression and wellbeing	Moderate
Köhler et al. (14)	Germany	Systematic review/meta-analysis	Multiple RCTs	Adult cancer patients	Significant effects on anxiety, depression, pain, fatigue	Moderate-High
Nyashanu et al. (8)	International	Scoping review	Multiple studies	Palliative care patients	Pain management, relaxation, spirituality, QoL as recurring themes	Moderate
Zhang et al. (17)	China	MCDM analysis	SOL data	Palliative patients	Confirmed improvement in QoL, spiritual wellbeing, ego-integrity, distress	Moderate
Brungardt et al. (13)	USA	Pilot study	NR	Palliative inpatients	VR-based MT: moderate symptom distress improvement; small existential QoL gains	Low
Jodie et al. (36)	International	Systematic review	Multiple studies	Palliative care patients	MT, aromatherapy, massage all showed short-term symptom benefits	Moderate
Pérez-Eizaguirre and Vergara-Moragues (37)	International	Systematic review	Multiple studies	Palliative patients	Pain, relaxation, happiness, reduced anxiety/depression, spirituality, QoL as themes	Moderate
Whitehead (1)	USA	Commentary	N/A	Oncology/palliative patients	MT among most frequently used complementary therapies in US/Canadian palliative programs	Low
Potvin et al. (28)	USA	Theoretical model	N/A	Informal hospice caregivers	Resource-oriented MT model for pre-bereavement caregiver support	Low-Moderate
Potvin et al. (25)	USA	Conceptual paper	N/A	Hospice/palliative programs	MT as core hospice service; argues for required counseling service positioning	Low
Gillespie et al. (7)	International	Mixed-methods systematic review	34 studies	Informal carers, pre/post-bereavement	Equivocal quant outcomes; moderate-strong qual support for psychosocial outcomes	Moderate-High
Whitford et al. (29)	USA	Feasibility study	NR	Caregivers at end of life	High caregiver satisfaction; stress scores worsened reflecting disease trajectory	Low-Moderate
Prieto-Crespo et al. (20)	International	Systematic review	Multiple studies	Palliative patients	Music and dance as spiritual interventions contributing to peace and acceptance	Moderate
Jaman-Mewes et al. (19)	International	Scoping review	Multiple studies	Palliative patients	MT frequently used alongside chaplaincy; complementary not interchangeable	Moderate
Kammin et al. (30)	International	Systematic review/qual synthesis	Multiple studies	Pediatric palliative	MT offered reprieve, normalized childhood, enhanced family wellbeing	Moderate
Overa (31)	International	Scoping review	Multiple studies	Pediatric palliative	Songwriting, improvisation, receptive listening documented across settings	Moderate
Bogacik et al. (38)	USA	Case study	*n* = 1	Pediatric palliative, home-based	MT and massage co-treatment supported comfort and family presence	Low
Van der Steen et al. (32)	International	Cochrane review	Multiple RCTs	People with dementia	Benefits for agitation, mood, QoL; effect sizes vary; quality mixed	High
Hu and Xu (33)	International	Systematic review/meta-analysis	Multiple RCTs	Older adults with dementia	Significant agitation reductions; supports MT as first-line non-pharmacological intervention	Moderate-High
Reidy and MacDonald (26)	Canada	Case report	NR	Palliative patients, COVID-19 context	Adapted MT delivery maintained therapeutic access during restricted visitation	Low
Baroni et al. (27)	Italy	National survey	Multiple hospices	Hospice programs	MT increasingly integrated into formal palliative care teams internationally	Low-Moderate
Wong et al. (34)	Singapore	Qualitative	NR	Gynae-oncology palliative patients	Culturally sensitive MT enabled grief processing, symptom relief in 1–2 sessions	Low-Moderate
Ramesh (35)	India	Narrative review	N/A	Indian palliative contexts	Western protocols require adaptation; calls for culturally grounded MT practice	Low
Koelsch and Bradt (22)	International	Narrative review	N/A	General/palliative	Music engages reward, emotion regulation, pain-modulating brain circuits	Moderate
Giordano et al. (23)	Italy	Case series	NR	Pediatric palliative	Songwriting creates lasting gifts for families; vehicle for emotional expression	Low
Valenti et al. (24)	USA	Mixed methods	NR	Acute palliative caregivers	Legacy MT intervention produced meaningful connection and meaning-making	Low-Moderate

NR, not reported.

## 3 Results

Table 1 presents a summary of all 33 included studies with quality ratings.

### 3.1 Psychosocial outcomes of music therapy

Across multiple randomized controlled trials and quantitative studies, music therapy consistently improves pain, distress, relaxation, and aspects of quality of life in palliative settings. A large U.S. RCT (*n* = 200) demonstrated that a single session of live music therapy with autogenic relaxation significantly reduced pain on numeric rating scales and functional pain scores compared with standard care alone (10). A German RCT with two live-music relaxation sessions improved relaxation, wellbeing, and fatigue and increased high-frequency heart rate variability, although pain did not differ significantly from a verbal relaxation control, suggesting that pain effects may depend on intervention type and comparator (11). A mixed-methods study in two U.S. hospitals found that live music reduced pain, anxiety, depression, nausea, and dyspnea while increasing wellbeing (12). A virtual reality-based music therapy pilot in an inpatient palliative consult service reported moderate improvements in total, physical, and psychological symptom distress and small gains in overall and existential quality of life (13).

Systematic review and meta-analytic evidence broadly supports these findings but with important caveats. The foundational Cochrane review by Bradt and Dileo (6), rated High quality, identified beneficial effects on pain and anxiety but concluded that all included trials had significant methodological limitations including high risk of bias, small sample sizes, and inadequate blinding. McConnell et al.'s (4, 5) updated systematic review of RCTs similarly found that music therapy significantly reduced pain but did not demonstrate improvements in global quality of life, and noted that the evidence base remained small and at high risk of bias. A broader systematic review and meta-analysis of music therapy in adult cancer patients found significant effects on anxiety, depression, pain, and fatigue (14), while Nyashanu et al.'s (8) scoping review identified pain management, relaxation, enhanced spirituality, and improved quality of life as recurring themes, though they also highlighted a lack of evidence on music therapy as a coping strategy for difficult conversations. The convergence across study designs is meaningful, but effect sizes are modest and confidence intervals wide, reflecting the predominantly small-sample, high-bias character of the evidence base.

Null and negative findings deserve explicit acknowledgment. Warth et al.'s (11) RCT found no significant pain reduction compared to verbal relaxation despite improvements in other outcomes. McConnell et al.'s (4, 5) systematic review found no evidence of quality of life improvement. Brungardt et al.'s (13) VR-based pilot found only small gains in existential quality of life. These null findings are not evidence that music therapy is ineffective, but they indicate that the field cannot yet reliably predict which outcomes will respond to which interventions in which populations.

### 3.2 Spiritual and existential wellbeing

Targeted biographical music therapy can meaningfully influence spiritual and existential domains, which are often the most difficult to address through conventional clinical means. The multicenter Song of Life (SOL) RCT, a three-session intervention built around a biographically meaningful song, did not change global or psychological quality of life but significantly improved spiritual wellbeing and ego-integrity and reduced momentary distress vs. a relaxation control, with high patient and family ratings of meaningfulness and importance (15). This multicenter RCT is rated High quality and represents the strongest single evidence source for music therapy's spiritual and existential effects. The SOL protocol explicitly frames music therapy around emotional and spiritual needs, targeting spiritual wellbeing, ego-integrity, distress, and psychobiological stress markers including cortisol (16). A multi-criteria evaluation applying entropy weighting and TOPSIS modeling to SOL data confirmed overall improvement in quality of life, spiritual wellbeing, ego-integrity, and distress (17).

A broader meta-analysis of brief psychosocial interventions found that interventions including music therapy and life review produced small to moderate improvements in quality of life and reductions in emotional and existential distress among palliative patients (18). A systematic review of spiritual care interventions in palliative settings identified music therapy as one of several interventions contributing to spiritual wellbeing, noting that music therapy was frequently used alongside chaplaincy rather than as a substitute for it (19). Importantly, the spiritual benefits of music therapy appear to operate through distinct mechanisms from chaplaincy: while chaplains provide explicitly religious and existential support through conversation, presence, and prayer, music therapy achieves spiritual outcomes through biographical engagement, emotional catharsis, and the non-verbal resonance of personally meaningful music (19, 20). Studies that have examined both suggest their effects are complementary rather than interchangeable, and that interprofessional models integrating both music therapy and spiritual care may offer advantages over either alone, though direct comparative evidence remains limited.

### 3.3 Mechanisms and theoretical models

A realist review of 51 articles maps how music therapy alleviates physical, psychological, emotional, and spiritual suffering in palliative care, identifying key contextual mechanisms including the therapeutic relationship, individualized music selection, and flexible use of receptive and active methods (3). The review emphasizes that research must move beyond outcome measurement to examine how and under what circumstances music therapy produces change. An integrative review combining patient and provider perspectives identifies an overarching theme of psychophysiological change, with patients linking music therapy to both positive and difficult emotional expression and increased wellbeing (2). The SOL psychoneuroendocrinological substudy explicitly conceptualizes music therapy as a psychobiological intervention: both music therapy and mindfulness led to decreased cortisol and mean heart rate over time, but only music therapy produced a significantly greater reduction in subjective distress (21). A neuroscientific review further details how music engages reward, emotional regulation, and pain-modulating brain circuits (22).

Together, these data support a model in which music therapy operates through multiple parallel pathways: emotional regulation and catharsis, biographical integration and meaning-making, neurobiological modulation of pain and stress, relational connection through the therapeutic relationship, and spiritual engagement through personally significant musical experience. The quality of evidence for these mechanisms varies substantially. Neurobiological mechanisms are supported by a growing translational science base (21, 22), though most mechanistic studies are small. Biographical and spiritual mechanisms are supported primarily by qualitative and mixed-methods evidence (2, 15) with moderate strength. The therapeutic relationship as a mechanism is theoretically compelling but has not been systematically measured or isolated in controlled studies.

### 3.4 Active, receptive, and legacy-oriented modalities

Music therapy in palliative care encompasses a spectrum of approaches. In the Gutgsell et al. (10) RCT, the intervention combined therapist-guided autogenic relaxation with live music performance. The German RCT used live music-based relaxation across two structured sessions (11). The SOL intervention adds a biographical dimension: patients select a personally meaningful song, the therapist explores the life stories connected to it, creates a recorded version, and shapes sessions as a form of musical life review and legacy, targeting psycho-spiritual integration (15, 16). Songwriting as a specific modality has received focused attention in both adult and pediatric palliative contexts (23, 24). The virtual reality-based pilot introduces a technology-mediated receptive modality with preliminary evidence for symptom distress and existential quality of life improvements (13).

No study has directly compared active vs. receptive modalities in a head-to-head trial within palliative populations, making it impossible to determine which approach is superior for which outcomes. The existing evidence suggests that biographical and legacy-oriented approaches may be particularly well-suited to spiritual and existential outcomes (15), while receptive and relaxation-focused approaches may be more effective for immediate symptom management (10, 11). Individualization appears to be a consistent principle across all effective modalities.

### 3.5 Canadian and North American contexts

Music therapy is among the most frequently used complementary therapies in inpatient palliative programs in the United States and Canada (1). The U.S. RCT by Gutgsell et al. (10) remains one of the strongest single studies supporting music therapy's effectiveness for pain in a North American context. A mixed-methods project in two U.S. hospitals documented significant symptom reductions, decreased opioid use trends, and strong patient and family narratives of benefit (12). Potvin et al.'s (25) conceptual framework for music therapy and nursing cotreatment describes how music therapy functions as a core hospice service in U.S. integrative programs and argues for its positioning as a required counseling service. The use of music therapy in Canadian palliative care was documented during the COVID-19 pandemic, when adapted delivery maintained therapeutic access during restricted visitation (26). A national survey of music therapy in Italian hospices illustrates the international trajectory toward formal integration, a trajectory that Canadian programs are increasingly mirroring (27).

### 3.6 Family, caregiver, and bereavement outcomes

The benefits of music therapy in palliative care extend beyond the patient to family members and informal caregivers. In the SOL trial, both patients and family members rated the intervention significantly more highly than the relaxation control, with large between-group effects for perceived meaningfulness and importance (15). A theoretical model of resource-oriented music therapy with informal hospice caregivers frames music therapy as a vehicle for rebuilding caregiver resources during the pre-bereavement phase (28). A mixed-methods systematic review of music therapy with informal carers pre- and post-bereavement (34 studies) found equivocal quantitative outcomes for quality of life and mental wellbeing, but moderate to strong qualitative and mixed-methods evidence for improved psychological, spiritual, emotional, and social outcomes (7). A feasibility study targeting caregiver distress at end of life found high satisfaction among caregivers and perceived benefit in stress relief, though caregiver stress scores worsened over time, likely reflecting the disease trajectory rather than intervention failure (29).

The quality of caregiver-focused evidence is notably weaker than patient-focused evidence. The systematic review by Gillespie et al. (7) documents substantial heterogeneity in designs and outcomes, with most studies relying on qualitative methods and few employing validated outcome instruments. The absence of randomized evidence specifically targeting caregiver outcomes represents an important gap.

### 3.7 Pediatric and dementia populations

Music therapy in pediatric palliative care presents distinct clinical and ethical considerations. A systematic review and qualitative evidence synthesis found that music therapy offered emotional and physical reprieve, normalized experiences of childhood within the clinical environment, enabled thriving despite serious illness, and enhanced family wellbeing (30). A scoping review documented the use of songwriting, improvisation, receptive listening, and family-integrated approaches across hospital, hospice, and home settings (31). Evidence quality in pediatric palliative music therapy is predominantly qualitative and case-based, with no RCTs identified in the literature.

In dementia care, Cochrane systematic review evidence supports music-based therapeutic interventions with benefits for agitation, mood, and quality of life, though effect sizes vary and study quality remains mixed (32). A systematic review and meta-analysis of music therapy for agitation in older adults dementia patients found significant reductions in agitation (33). The Cochrane review is the strongest evidence in this domain, with a large number of included trials, though substantial heterogeneity limits conclusions about which specific approaches work best.

### 3.8 Non-Western and culturally diverse contexts

The overwhelming majority of palliative music therapy research originates from North America, Europe, and Australia. Searches of English-language indexed databases conducted for this review identified only one study specifically from China, two from Japan, and two from India addressing music therapy in palliative contexts. This near-total absence in English-language indexed literature reflects a significant gap in accessible research rather than an absolute absence of music therapy practice or research in non-Western contexts; it is likely that relevant literature exists in Chinese, Japanese, Korean, Hindi, and other languages that was not captured by the English-language search strategy applied here.

The limited available English-language evidence is instructive. A qualitative study of inpatient music therapy with women receiving gynecological oncology palliative care in Singapore found that culturally sensitive, reflexive practice was essential to building trust and enabling patients to express appreciation of life, process grief, and experience symptom relief within one to two sessions (34). A narrative review of music therapy in Indian palliative care contexts calls for the development of culturally grounded practice within a biopsychosocial and salutogenic model, explicitly noting that Western intervention protocols require substantial adaptation for Indian cultural and religious contexts (35).

The role of religion in palliative music therapy represents a particularly underexplored area in the English-language literature. In many non-Western contexts, spiritual and religious dimensions of dying are inseparable from cultural identity, and music therapy's effectiveness may depend on whether the music and approach selected align with patients' religious frameworks. In Islamic contexts, the appropriateness of music in religious practice varies across traditions and may require careful navigation. In Buddhist contexts, music associated with impermanence, acceptance, and peaceful transition may carry particular resonance. In African cultural contexts, music's role in communal mourning rituals may suggest that group-based rather than individual music therapy models are more culturally congruent. Systematic reviews on carer-focused music therapy and spiritual support both note the absence of English-language research from low- and middle-income countries and call for culturally adapted interventions globally (7, 20).

### 3.9 Reporting quality and evidence gaps

The palliative music therapy evidence base is characterized by significant methodological heterogeneity and persistent limitations. The foundational Cochrane review by Bradt and Dileo (6) identified that all included RCTs had significant methodological limitations. McConnell et al.'s (4, 5) updated systematic review found that all trials had high risk of bias. Systematic reviews consistently identify heterogeneity in outcome measures, session numbers, therapist training, and intervention formats as barriers to synthesis and evidence accumulation (2, 3, 8). The realist review calls for research that moves beyond effectiveness measurement to systematically test theories of change and implementation (3). Workforce shortages, lack of reimbursement, and the ethical and logistical complexities of conducting rigorous research with terminally ill populations remain underexplored barriers to implementation (25, 35).

## 4 Discussion

The reviewed literature consistently supports music therapy as a holistic, flexible, and low-risk intervention for patients in palliative and end-of-life care. Evidence is strongest for improvements in pain, mood, anxiety, and subjective wellbeing, outcomes of high clinical relevance given the distressing nature of terminal illness (10–12, 14). Biographical and legacy-oriented approaches demonstrate capacity to address the spiritual and existential dimensions of dying that standard clinical care often struggles to reach (15, 17, 18). Families and informal caregivers also benefit, particularly through shared musical experiences, legacy creation, and pre-bereavement psychosocial support (7, 28, 29).

A particularly meaningful theoretical contribution from the literature is the recognition that music therapy operates through multiple simultaneous mechanisms, neurobiological, emotional, biographical, relational, and spiritual, that reinforce one another (3, 21, 22). This multimechanistic quality distinguishes music therapy from pharmacological approaches and positions it as a genuinely integrative intervention capable of addressing the multidimensional suffering that characterizes advanced illness.

The limitations of the current evidence base are substantial. Most studies are small, non-randomized, or at high risk of bias; effect sizes are modest; outcome measures vary widely; and research is heavily concentrated in Western, high-income settings (4–7). The field urgently needs large-scale, culturally diverse, mixed-methods trials with standardized outcome sets to support evidence-based integration of music therapy into palliative care systems globally. The near-total absence of palliative music therapy research in English-language indexed databases from Asian, African, and other non-Western contexts, documented systematically in this review, represents one of the field's most significant gaps and a call for both multilingual searches and non-Western primary research in future syntheses.

## 5 Conclusion

Music therapy offers significant psychosocial benefits for patients in palliative and end-of-life care, including improved mood and emotional wellbeing, reduced pain, anxiety, and fatigue, enhanced spiritual wellbeing and ego-integrity, and opportunities for legacy-building and meaning-making that standard clinical care rarely provides. Evidence also supports meaningful pre-bereavement benefits for family members and informal caregivers through shared musical experience, relational connection, and legacy creation. These benefits are observed across adult, pediatric, and dementia populations and across active, receptive, and biographical modalities.

However, the evidence base remains constrained by small samples, high risk of bias, heterogeneous designs, and critical gaps around long-term outcomes, cultural adaptation, and non-Western contexts. To fully realize music therapy's potential as a standard component of palliative care, future research should: (1) prioritize large-scale, culturally sensitive randomized trials; (2) adopt standardized outcome sets encompassing physical, psychological, spiritual, and caregiver domains; (3) systematically investigate implementation barriers including workforce development, reimbursement, and interprofessional integration with chaplaincy and other spiritual care providers; (4) develop and evaluate culturally adapted music therapy models for diverse religious and ethnic populations, particularly in Asian, African, and other non-Western settings; (5) directly compare active vs. receptive modalities; and (6) conduct multilingual searches in future systematic reviews to capture non-Western literature currently excluded by English-language search strategies.
